# Five years experience on 3,4-diaminopyridine phosphate in Lambert–Eaton syndrome: Case reports

**DOI:** 10.1097/MD.0000000000007839

**Published:** 2017-09-22

**Authors:** Simona Portaro, Teresa Brizzi, Stefano Sinicropi, Alberto Cacciola, Maria Cristina De Cola, Alessia Bramanti, Demetrio Milardi, Antonino Lupica, Placido Bramanti, Antonio Toscano, Carmelo Rodolico

**Affiliations:** a IRCSS Centro Neurolesi “Bonino-Pulejo”, Neuromuscular Disease Laboratory; b Department of Clinical and Experimental Medicine, University of Messina, Messina; c DIBIMIS, University of Palermo, Palermo, Italy.

**Keywords:** 3,4-diaminopyridine phosphate, nonparaneoplastic-Lambert–Eaton myasthenic syndrome

## Abstract

**Rationale:**

To report our experience on 7 patients (4 males and 3 females), affected by nonparaneoplastic Lambert–Eaton myasthenic syndrome, treated with 3,4-diaminopyridine phosphate (3,4-DAPP) either alone or in combination with other immunosuppressants or steroids.

**Patient concerns:**

Patients have been evaluated at specific timepoints (ie, baseline and last 5 year follow-up), with neurological examination, autoantibodies against presynaptic voltage-gated Cav2.1 (P/Q type) calcium ion channel (VGCC) dosage, neurophysiological evaluation focusing on the increased amplitude of the compound muscle action potential (cMAP) after maximum voluntary effort, quantitative myasthenia gravis (QMG) and activities of daily living scales, and autonomic nervous system involvement evaluation.

**Outcomes:**

Five out of 7 patients presented a clinical improvement persisting at last 5-year follow-up; 2 out of them improved taking only 3,4-DAPP at the maximal dosage, whereas the remaining received concomitant medications, such as prednisone and azathioprine. However, the clinical amelioration was not statistically significant. No one of the patients reported severe adverse events, except one, complaining of transient chin and perioral paresthesias. A significant association between QMG and the type of pharmacological drugs therapy (*P* = .028) emerged. Indeed, we observed an improvement of the clinical condition in all 3 subjects treated with 3,4-DAPP and prednisone.

**Conclusions:**

In this study, we confirm 3,4-DAPP treatment efficacy on muscle strength, but minor evidence of drug effectiveness have been demonstrated on the autonomic nervous system involvement and on the deep tendon reflexes reappearance, a part from patients who received 3,4-DAPP associated to prednisone.

## 1 Introduction

Lambert–Eaton myasthenic syndrome (LEMS) is a rare autoimmune disorder caused by autoantibodies against presynaptic voltage-gated Cav2.1 (P/Q type) calcium ion channel (VGCC) at the neuromuscular junction, causing a decrease of calcium influx that prevents the release of acetylcholine (ACh) from the nerve terminals and attenuates normal muscle contraction.^[1–5]^ LEMS is estimated to affect 1:100,000 people in the European community, with an incidence of 0.48 to 0.75 per million,^[6]^ and to have variable clinical onset, ranging from 20 to 50 years of age,^[7]^ even though childhood and infantile forms have been reported.^[8–14]^ It is characterized by limb girdle muscles weakness, easy fatigability, absent deep tendon reflexes with posttetanic potentiation, and autonomic alterations, such as dry mouth, constipation, and erectile dysfunction. Activities associated with daily functioning, such as climbing stairs, rising from a chair, health, and self-care management, are involved as well.^[15]^ It has been widely demonstrated in biopsied intercostal muscles that reduced quantal release of ACh plays a key role in the pathophysiology of LEMS.^[16,17]^ Moreover, strong evidences suggest an antibody mediated mechanism.^[18,19]^ The disorder can be either paraneoplastic (P-LEMS)^[20]^ or associated with autoimmune disorders (nonparaneoplastic Lambert–Eaton myasthenic syndrome [NP-LEMS]).^[21,22]^ Antibodies to P/Q-type VGCCs can be detected in over 90% of both NP- and P-LEMS, since they are specific for the disorder.^[23]^

LEMS diagnosis is based on the suggestive clinical presentation (proximal weakness, absence of tendon reflexes, and signs of autonomic dysfunction), immunological testing (anti-VGCC antibody assay), and electrophysiological studies (showing a presynaptic defect of the neuromuscular transmission).^[24]^

The neurophysiologic study reveals a presynaptic neuromuscular junction impairment with reduced amplitude of compound muscle action potential (cMAP) at rest; the cMAP amplitude decreases during low-rate (2–5 Hz) repetitive nerve stimulation (RNS) and increases by more than 100% after maximum voluntary activation or after 50 Hz nerve stimulation.^[20,24–28]^ The presence of antibodies against Cav2.1 P/Q-type VGCC in serum further supports the diagnosis.^[1]^

During the last decade, several symptomatic treatments, such as pyridostigmine, guanidine, 4-aminopyridine, and 3,4-diaminopyridine (3,4-DAP), have been tried, but only aminopyridines were found to be the most effective.^[29]^ In fact, aminopyridines enhance the release of ACh from the motor nerve terminal thus improving neuromuscular transmission by blocking voltage-activated K+ channels. Among these molecules, 4-aminopyridine produces marked improvement in muscle strength in LEMS patients, but its clinical use is limited since it triggers seizures at therapeutic doses.^[30,31]^ Unlike other aminopyridines, 3,4-diaminopyridine phosphate (3,4-DAPP) has limited penetration into the brain and thus leads to few central nervous system side effects.^[32]^ Indeed, it has been reported that side effects, including seizures, occurred less frequently^[33]^ and the risk of seizures appears to be dose-dependent.^[34]^ Consequently, 3,4-DAPP has been used to treat patients with LEMS for over 20 years in Europe, and the reported experience consistently indicates that 3,4-DAPP is a safe, effective, and valuable treatment for LEMS.^[32,35]^ Although 3,4-DAP base has only been available via named-patient programmes, requiring ad hoc preparations in compounding pharmacies, tablets containing 3,4-DAP phosphate salt, equivalent to 10 mg base, have become available. This formulation has obtained the orphan medicinal product status both in the European Union and in the United States of America and has received marketing authorization in Europe as Firdapse. These tablets have been shown to be essentially bioequivalent with the base preparation.^[33]^ A recent study on veterans affair population showed that patients treated with 3,4-DAP had the highest percentage of clinical improvement or resolution (78%, based upon clinical exam).^[36]^

Herein, we report our clinical experience on 7 NP-LEMS patients treated with 3,4-DAPP either alone or in combination with other immunosuppressants or steroids.

## 2 Materials and methods

### 2.1 Patients

Seven patients (mean age: 50.3 ± 10.2 years; 57.1% males) affected by LEMS, attending our clinic from 2009 to date, were evaluated in this retrospective study. Baseline (T_0_) and 5-year follow-up (T_1_) data are reported in Tables 1 and 2, respectively. LEMS diagnosis was made if the following conditions were present: weakness that predominated in proximal limb muscles; electroneurographic findings characteristic of LEMS: small cMAPs increasing at least 2-fold after maximum voluntary contraction of the tested muscle (abductor digiti minimi); and presence of autoantibodies against presynaptic VGCC, as supportive data to reinforce the diagnosis. We retrospectively analyzed 5 years data using LEMS registry worksheet which consists of clinical evaluation, Quantitative Myasthenia Gravis (QMG) score, dedicated functional scale to evaluate activities associated with daily functioning, neurophysiological evaluation, and VGCC dosage. All patients were evaluated twice a year for 5 years. The study was approved by local ethics committees and conducted in accordance with the Declaration of Helsinki (1975), revised Hong Kong (1989).

**Table 1:**
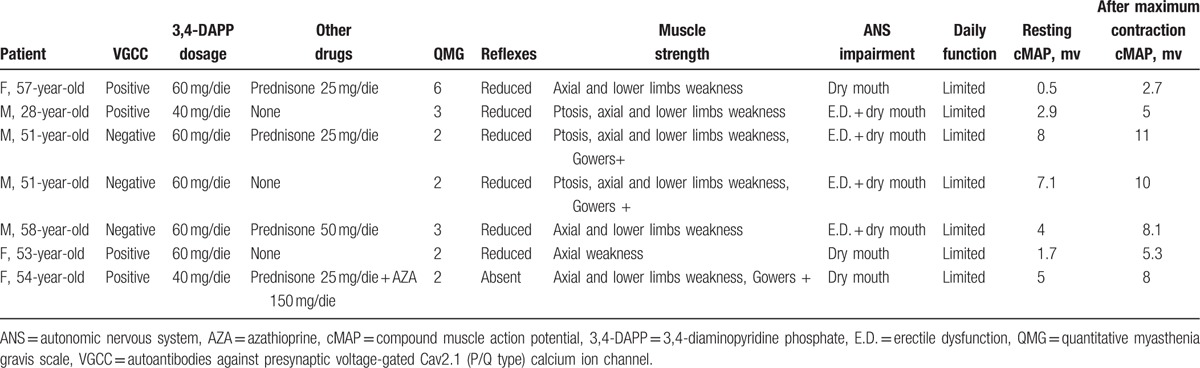
Patients’ clinical data at baseline.

**Table 2:**
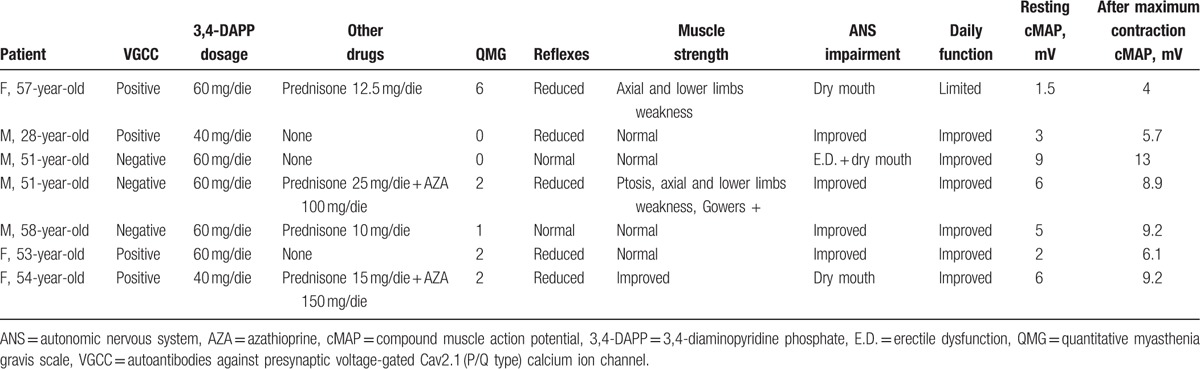
Patients’ clinical data at follow-up.

### 2.2 Clinical assessment

Patients underwent a neurological examination performed by a neurologist skilled on neuromuscular disorders, applying the 5 point-Medical Research Council scale and the QMG scale. All patients were screened using the delta-P score^[37]^ and a thorax CT scan in order to disclose a small cell lung cancer. Only 1 patient was a smoker. The presence of autonomic nervous system involvement was investigated through a simple questionnaire, which was part of the LEMS Registry Worksheet, investigating for the presence of erectile dysfunction in males and for dry mouth in both sexes. All patients started therapy with 3,4-DAPP at therapeutic dosage, during the 5-year follow-up, alone or in addition or replacing immunosuppressive medications. Antibodies assays the related VGCC antibodies were tested using commercially available kits to assay Cav2.1 P/Qtype VGCC autoantibody titers.

### 2.3 Electrophysiological evaluation

Electrophysiological assessment was performed according to a standardized assessment protocol specifically for the evaluation of LEMS.^[24–26]^ The cMAP amplitude obtained from electroneurography was measured in mV, following RNS at rates of 3 to 5 Hz, searching for a decrementing pattern of greater than 10% in the 4th or 5th amplitude response following RNS.^[24–26]^ cMAP amplitude after maximum voluntary contraction has been evaluated on the abductor digiti minimi for each patient^[29]^ at diagnosis and at follow-up. The evaluation of cMAP amplitude after maximum voluntary contraction is the technique of choice as it is better tolerated instead of high frequency (50 Hz) RNS that is very painful and not well tolerated by patients.^[24]^

### 2.4 Statistical analysis

Statistical analysis was performed by using the 3.2.3 version of the open-source software R, by setting *P* < .05 as significance level.^[38]^ To compare cMAP amplitude between T_0_ and T_1_ and the clinical evaluation scores of QMG between patients treated only with 3,4-DAPP and 3,4-DAPP in association with other drugs (both at T_0_ and T_1_) we used the Mann–Whitney *U* test. Most of the qualitative variables (ie, reflexes, muscle strength, autonomic nervous system, and daily function) were converted into binary variables as follows: 1 if the patients showed an improvement from T_0_ to T_1_; 0 if the clinical situation of the patients was stable. The Fisher exact test was used to compare proportions in contingence tables.

## 3 Results

**Figure 1:**
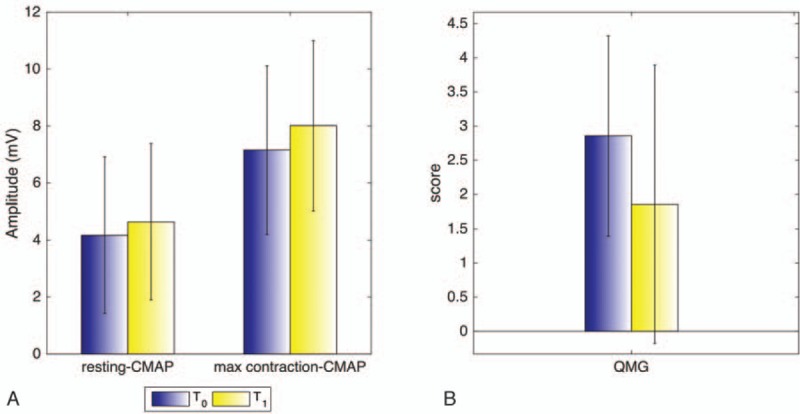
Barplots show differences between T_0_ and T_1_ for resting state cMAP amplitude and after maximum voluntary contraction (A) and for QMG scores (B). No significant differences were found (*P* > .05). Error bars refer to SD. cMAP = compound muscle action potential, QMG = quantitative myasthenia gravis scale, SD = standard deviation.

Four out of 7 patients (57.1%) resulted VGCC positive. The average QMG score at T_0_ was 2.86 (median = 2; min = 2; max = 6), whereas at T_1_ was 1.86 (median = 2; min = 0; max = 6). Although a slight improvement was evident, such a difference was not statistically significant (*P* = .17) (Fig. 1). No significant difference in QMG scores between patients treated only with 3,4-DAPP and those treated with 3,4-DAPP in association with other drugs was found, neither at T_0_ nor at T_1_. However, the clinical condition of all 3 subjects treated with 3,4-DAPP in association with Prednisone improved. Indeed, the QMG score of these patients increased of at least 2 point. The Fisher exact test did not detect any significant association between improvement in reflexes and VGCC, as well as the daily dosage of 3,4-DAPP, or the presence of other drugs in therapy. Similar results were found on improvement in muscle strength, autonomic nervous system, and daily function. The electrophysiological findings did not disclose any significant variation in the cMAP amplitude at the 5-year follow-up assessment (Fig. 1).

## 4 Discussion

LEMS is a rare and autoimmune disorder of neuromuscular transmission with presynaptic involvement.^[16]^ Abnormal electrophysiological findings, along with the presence of elevated VGCC autoantibody titers, confirm the diagnosis.^[20,27]^ Herein, we retrospectively reported on the clinical findings of a small cohort of NP-LEMS patients with a mild-to-moderate neuromuscular impairment. The majority of patients were assessed as having reduced or limited functioning for daily activities, such as the ability to walk upstairs, cycle, arise from a low chair with and without arm support, arise from sitting on 1 knee or squatting, and climbing stairs with and without arm support. As previously reported, several studies demonstrated that patients with LEMS, treated with 3,4-DAP administration, exhibited significant improvements in muscle strength (Table 3).^[34,39,40]^ Recently, Mantegazza et al^[3]^ and Oh et al^[42]^ reported the same beneficial effects at approximatively the same 3,4-DAPP dosage (Table 3). Other studies showed that also 3,4-DAP base, but at higher dosage, led to similar results in some patients^[3,29,33,34,36,39,42]^ (Table 3). It is worthwhile to note that 3,4-DAP as the free base is available for LEMS patients only from compounding pharmacies in several EU countries. However, concern remains over the form of amifampridine prescribed since considerable variability has been observed in the active pharmaceutical ingredient content quantified from laboratory analyses of samples of compounded 3,4-DAP base.^[41]^ Consequently, the 3,4-DAPP is currently the only safe and approved 3,4-DAP compound for the symptomatic treatment of LEMS in adults in the EU.^[3,42]^ Our patients received 3,4-DAPP at diagnosis to improve neuromuscular performances (*P* > .05, Fig. 1), even though immunosuppressant agents were added in case of poor response. None of our patients underwent plasma exchange or immunoglobulin treatment during the follow-up period. Interestingly, patients who received prednisone in association with 3,4-DAPP manifested the posttetanic potentiation with the re-appearance of the deep tendon reflexes; 1 patient who reduced the initial dosage of prednisone had the appearance of weak deep tendon reflexes. The underlying mechanism that may explain this phenomenon is a possible add on treatment potentiation induced by prednisone. Indeed, this phenomenon was not disclosed in those patients who were treated only with 3,4-DAPP.

**Table 3:**
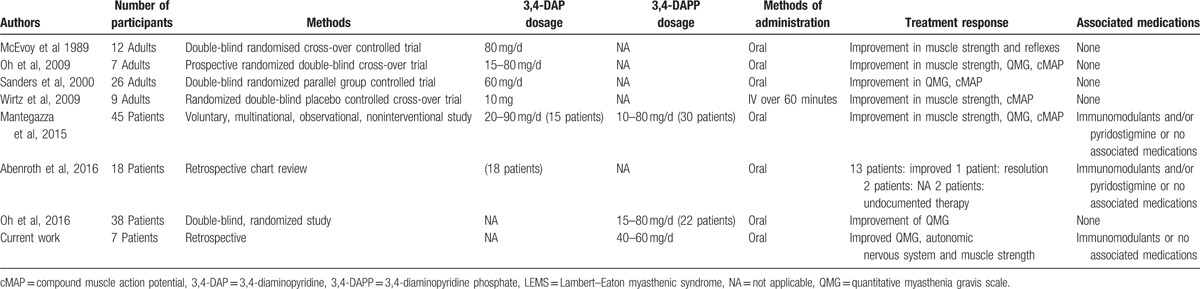
Paradigm on the use of 3,4-DAP and 3,4-DAPP in LEMS.

The present work has some limitations, such as the small sample size and the retrospective nature of the study that does not allow to a randomization. However, some of our patients data have been included in the LEMS patient registry, which was launched in the European community in mid-2010 as an observational, voluntary, multinational, noninterventional program to collect structured empirical data on clinical course, treatment utilization, and safety and efficacy from the use of LEMS-specific treatments.^[3]^ We herein stress the concept that LEMS patients assuming 3,4-DAPP reported a subjective consistent clinical improvement without significant side effects. However, further studies and randomized clinical trials including a bigger number of patients should be fostered to shed new light on the possible action mechanism of 3,4-DAPP and to confirm its effectiveness in LEMS.
